# Leveraging a Validated *in silico* Approach to Elucidate Genotype-Specific VP7 Epitopes and Antigenic Relationships of Porcine Rotavirus A

**DOI:** 10.3389/fgene.2020.00828

**Published:** 2020-07-31

**Authors:** Frances K. Shepherd, Cheryl M. T. Dvorak, Michael P. Murtaugh, Douglas G. Marthaler

**Affiliations:** ^1^Department of Veterinary and Biomedical Sciences, College of Veterinary Medicine, University of Minnesota, Saint Paul, MN, United States; ^2^Department of Diagnostic Medicine and Pathobiology, College of Veterinary Medicine, Kansas State University, Manhattan, KS, United States

**Keywords:** rotavirus A, B cell epitopes, immunoinformatics, bioinformatics, vaccine

## Abstract

Rotavirus A (RVA) remains one of the most widespread causes of diarrheal disease and mortality in piglets despite decades of research and efforts to boost lactogenic immunity for passive protection. Genetic changes at B cell epitopes (BCEs) may be driving failure of lactogenic immunity, which relies on production of IgA antibodies to passively neutralize RVA within the piglet gut, yet little research has mapped epitopes to swine-specific strains of RVA. Here we describe a bioinformatic approach to predict BCEs on the VP7 outer capsid protein using sequence data alone. We first validated the approach using a previously published dataset of VP7-specific cross-neutralization titers, and found that amino acid changes at predicted BCEs on the VP7 protein allowed for accurate recapitulation of antigenic relationships among the strains. Applying the approach to a dataset of swine RVA sequences identified 9 of the 11 known BCEs previously mapped to swine strains, indicating that epitope prediction can identify sites that are known to drive neutralization escape *in vitro*. Additional genotype-specific BCEs were also predicted that may be the cause of antigenic differences among strains of RVA on farms and should be targeted for further confirmatory work. The results of this work lay the groundwork for high throughput, immunologically-relevant analysis of swine RVA sequence data, and provide potential sites that can be targeted with vaccines to reduce piglet mortality and support farm health.

## Introduction

Rotaviruses are double-stranded segmented RNA viruses in the *Reoviridae* family that are among the leading causes of diarrheal disease in a wide range of host species including swine (Estes and Greenberg, 2013; Shepherd et al., 2019). Rotavirus A (RVA) is the most common rotavirus species identified in pigs. The outer capsid viral protein 7 (VP7) and VP4 are the targets of neutralization and are used to designate G and P genotypes, respectively, based on sequence similarity of the encoding genes (Estes and Greenberg, 2013). Currently, 12 G and 16 P RVA genotypes have been identified pigs (Vlasova et al., 2017). High genetic diversity and potential for viral reassortment are significant challenges to reducing the burden of RVA disease on farms.

RVA infects the small intestinal epithelium and causes sloughing of the villi, leading to malabsorptive diarrhea which is most severe in young piglets. Since piglets are born agammaglobulinemic, passive lactogenic immunity is required to protect them from RVA morbidity and mortality (Hammerberg et al., 1989; Bianchi et al., 1999; Shepherd et al., 2019). Evidence suggests that some heterotypic protection can occur through stimulating active immunity with multiple exposures to one or several viral strains (Bishop et al., 1986; Chiba et al., 1993; Franco et al., 2006; Jiang et al., 2013; Nair et al., 2017), but it is not well understood whether passive immunity offers the same breadth of protection for piglets. Regardless, the most effective protection is observed in homotypic as opposed to heterotypic challenge (Gaul et al., 1982; Bishop et al., 1986; Hoshino et al., 1988), highlighting the importance of matching a vaccine VP7 and VP4 to the field strain of interest.

A modified live vaccine for porcine RVA (ProSystem RCE, Merck Animal Health) is licensed for commercial use in pregnant gilts and sows, but contains limited genotypic diversity and the rate of usage by veterinarians is unknown (Saif and Fernandez, 1996). Prefarrow natural planned exposure (NPE) is more commonly used to stimulate lactogenic immunity to farm-specific RV strains in the United States, but risks introducing unwanted pathogens into the farrowing room. Recently developed RNA particle (RP) vaccines have been used to vaccinate against swine influenza A virus and porcine epidemic diarrhea virus (Sandbulte et al., 2015; Kim et al., 2016), and could be applied to RV to reduce the use of live virus. An advantage of RP vaccines is their ability to be quickly updated to match new viral strains, but knowledge of genetic changes that drive immune escape will be crucial to understanding when vaccines should be changed. Given the importance of antibody-based immunity in RV infections, B cell epitopes on the RV VP7 and VP4 are likely important sites for developing resistance to vaccines and warrant further study.

Epitope mapping studies of RVA found that even one amino acid change can result in resistance to neutralization by monoclonal antibodies (mAbs) (Coulson and Kirkwood, 1991; Kang et al., 1993; Kirkwood et al., 1993; Ciarlet et al., 1994). However, classical identification of RV epitopes utilizing mAbs to find sites of neutralization escape mutations is time- and resource-intensive. Plus, this approach does not recapitulate the polyclonal immune response in a natural infection and may fail to comprehensively describe epitopic residues due to the random nature of mAb isolation. Moreover, available epitope research utilizing porcine RVA strains is limited to 4 of the 12 G genotypes identified in pigs and has not been described for G9 strains, the most common G genotype in swine (Aoki et al., 2009; Vlasova et al., 2017; Shepherd et al., 2019). Instead, *in silico* epitope prediction from genetic data provides a unique opportunity for high throughput, clinically relevant analysis of gene sequences. Here, we use bioinformatic tools to assess the agreement between *in vitro* neutralization data and B cell epitopes predicted using the EPCES method (Liang et al., 2009) in a test dataset of RVA strains (Hoshino et al., 2004). We then apply EPCES to a dataset of porcine RVA G3, G4, G5, G9, and G11 strains to identify potential antibody binding sites that could be targeted with recombinant vaccines.

## Materials and Methods

### Methodology Validation

The cross-neutralization behavior of RVA VP7 was previously described using human G9 strains and a bovine G6 strain (Hoshino et al., 2004). Briefly, Hoshino et al. (2004) reassorted the VP7 gene from nine human G9 strains (WI61, F45, AU32, 116E, R44, R143, US1205, INL1, and BD524) with the remaining 10 RVA genes from the bovine UK strain and generated hyperimmune sera in guinea pigs against the reassortant viruses (Hoshino et al., 2004). The serum was used to determine 60% plaque reduction neutralization (PRN) titers for ten human G9 strains (WI61, DS-1 x WI61 reassortant, F45, AU32, 116E, R44, R143, US1205, INL1, and BD524) and the UK G6 bovine strain (Hoshino et al., 2004). The DS-1 x WI61 reassortant containing the WI61 VP7 on a DS-1 backbone was used to identify if the VP4 gene from WI61 affected neutralization titers (Hoshino et al., 2004). The reassortant behaved similarly to the parental WI61 strain (Hoshino et al., 2004). Due to the use of a common backbone in the generation of hyperimmune sera, differences in PRN titers are assumed to be due to differences in the VP7 gene.

The PRN titers were mapped using antigenic cartography accessed at acmacs-web.antigenic-cartography.org (Smith, 2004; de Jong et al., 2007; Lorusso et al., 2011; Lewis et al., 2014; Abente et al., 2016). Antigenic cartography uses modified multidimensional scaling (MDS) methods to place strains and antisera in shape space, such that the difference between the log_2_ of the PRN titer and the Euclidean distance on the graph between a strain and a corresponding antisera is minimized (Smith, 2004). Map distances are defined by the equation log_2_(b_j_)-log_2_(N_ij_) with b_j_ indicating the minimum column basis, automatically adjusted in acmacs-web.antigenic-cartography.org, and N_ij_ representing the titer differences between serum *i* and antigen *J.* Two-, three-, and four-dimensional maps were created using 5000 random restart optimizations to determine the dimensionality that resulted in the lowest stress. Linear and loess regression were used to assess goodness of fit between table distances (PRN titers) and map distances. K-means clustering was used to determine antigenic clusters. A substitution at site L from some amino acid X to a different amino acid Y was considered a cluster defining mutation (CDM) between two clusters A and B if at least 80% of strains in cluster A have amino acid X, and at least 80% of strains in cluster B have amino acid Y (Smith, 2004; Koel et al., 2013).

Additional maps of the RVA test set were created using MDS in R version 3.6.0 (R Core Team, 2018) where the distances among pairs of strains represent the number of amino acid substitutions. The goal was to determine whether putative antigenic clusters, based on amino acid diversity, matched the clusters identified in the antigenic maps, which are based on neutralization titers. The VP7 amino acid sequences were aligned (the output is referred to as “original alignment” below) using MAFFT in Geneious Prime (version 2019.2.3, Biomatters^1^). The original alignment was then edited in three ways using the “Mask Alignment” tool in Geneious Prime which removes specified residue positions from an alignment of sequences and concatenates the remaining positions. First, the conserved residues were removed from the original alignment to look at all variable positions within the VP7 gene. This was performed by using the “Mask Alignment” tool with the “sites containing identical residues” option. Second, the original alignment was reduced to only surface-exposed residues on the outward-facing surface of the VP7 protein to look at all possible B cell epitopes. This was performed using the Mask Alignment tool with the NEXUS-style CHARSET option. Surface-exposed residues were determined using a crystallized structure of VP7 (PDB #4v7q) and identifying all residues with a solvent accessible surface area of greater than 2.5 angstroms^2^ in UCSF Chimera (Pettersen et al., 2004). Residues that were exposed, but on surfaces inaccessible to antibodies (i.e., on the inner-facing surface of the VP7) were identified through visualizing the VP7 trimer protein structure in Chimera and were not considered for this alignment. Finally, the original alignment was reduced to only the predicted B cell epitopes (pBCEs) as calculated below, again using the NEXUS-style CHARSET option. Pairwise amino acid distances were calculated in Geneious Prime from the three types of additional alignments. Genetic clusters were defined using k-means clustering in R (R Core Team, 2018). Only WI61 is described in the genetic cluster analyses since WI61 and DS-1 x WI61 share the same VP7 gene.

Prediction of conformational BCEs was carried out using EPCES (Liang et al., 2009). Homology modeling in SWISS-MODEL (Arnold et al., 2006) generated the putative protein structures of the RVA VP7 sequences. The VP7 of RVA strain RRV has been crystallized in a bound (PDB ID #3fmg) and unbound (PDB ID #4v7q) state (Aoki et al., 2009; Settembre et al., 2011), and each structure was used for homology modeling to identify the best template (bound vs. unbound) for BCE prediction. PDB ID #4v7q was chosen as the unbound template because it yielded the highest QMEAN and GQME scores (measures of model quality) during the SWISS-MODEL modeling process. The EPCES scores from the individual human G9 strains were averaged at each residue in the sequence to identify high-scoring residues within the G9 genotype. The UK G6 strain was separately scored to identify genotype-specific pBCEs within G6. Genotype-specific pBCEs were determined by identifying the surface exposed residues which were in the top 15% of scores (Liang et al., 2009). The unbound VP7 structure generated homology models with an N-terminal tail that was assigned an artificially high score by EPCES. Therefore, we excluded any pBCEs that would be biologically implausible, such as pBCEs on the N terminus. Predicted BCEs were assigned patches based on geometric distance, and an assumed average antibody binding footprint of 1100 Å^2^ (Ramaraj et al., 2012).

### B Cell Epitope Prediction of Porcine RVA VP7 and Identification of Putative Antigenic Clusters

Porcine RVA VP7 sequences were obtained from the University of Minnesota Veterinary Diagnostic Laboratory (UMN-VDL) from intestinal and fecal samples from pigs that were routinely received for diagnostic testing. Sequences were obtained from select RVA-positive samples as determined by real time RT-qPCR in which the veterinarian requested sample sequencing (Marthaler et al., 2014). Between 2009 and 2014, 196 VP7 sequences were obtained at the UMN-VDL from pigs across the United States. Each strain was assigned a genotype based on having at least 80% nucleotide sequence identity with strains in GenBank using a BLAST search (Altschul et al., 1990; Matthijnssens et al., 2008). Sequences were submitted to GenBank under accession numbers MN862084-MN862279.

BCEs were predicted in the porcine VP7 dataset by homology modeling with the unbound crystallized structure (PDB ID #4v7q) as described above. Porcine sequences were grouped by genotype, and the average EPCES score per residue was calculated. The top 15% of EPCES scores at surface-exposed sites, determined as described above using PDB ID #4v7q, were determined to be pBCEs. The surface-exposed residues determined using 4v7q could be different than those present within the swine VP7 proteins, which may lead to incorrect epitope prediction. Therefore, the surface-exposed residues from 4v7q were compared to the surface-exposed residues from 5 representative homology models from each of the G3, G4, and G5 datasets, 10 representative G9 models, and 3 representative G11 models. The average EPCES score at any discrepant site (i.e., a surface-exposed residue from 4v7q that was buried within one of the representative models, or a buried site in 4v7q that was surface-exposed in a representative model) was investigated to determine whether any high-scoring residues were missed by using the 4v7q surface-exposed residues. We found that none of the discrepant sites had EPCES scores above 44, which was well below the low end of average EPCES scores (no lower than 65) for pBCEs determined using 4v7q surface-exposed residues. Therefore, the variation between surface-exposed residues in 4v7q and our VP7 strains did not affect BCE prediction.

To identify putative antigenic clusters within genotypes of porcine RVA VP7, we performed MDS of the pairwise amino acid differences at pBCEs in R. Clusters were identified using k-means clustering in R, and the number of clusters was chosen using lowest within-sum of squares and silhouette methods in the factoextra package in R (Kassambara and Mundt, 2017). In the G5 and G9 analyses, the Prosystem RCE vaccine strains A2 and OSU (Intervet, Inc., Merck Animal Health, Madison, NJ) were also included (GenBank accession numbers KR052761 and AB180971).

## Results

### Validation of B Cell Epitope Prediction

We first aimed to validate the biological relevance of *in silico* epitope predictions by determining whether amino acid variability at pBCEs could be predictive of antigenic distances among RVA strains. Antigenic maps were created using previously published PRN titers from a set of ten human RVA G9 strains and one bovine G6 strain (Hoshino et al., 2004). Mapping in 3-dimensions yielded the highest R^2^ and adjusted R^2^ (*R*^2^ = 0.90), and 0.06% reduction in stress compared to 2-dimensions (Supplementary Table 1 and Supplementary Figure 1), indicating neutralization titers were well-represented by the 3-dimensional antigenic map. Stress and R^2^ values did not change between 3 and 4 dimensions (Supplementary Table 1), so 3 dimensions were chosen for final antigenic cartography analysis.

**FIGURE 1:**
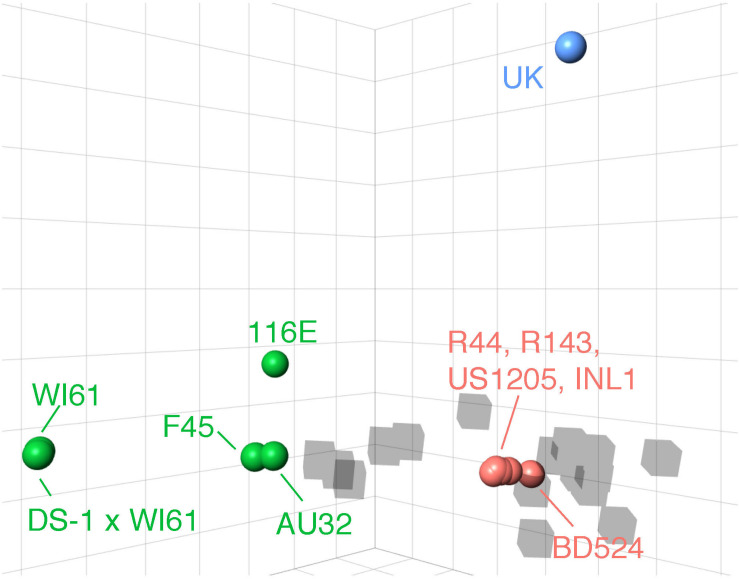
3D antigenic cartography of human G9 and bovine G6 RVA strains based on VP7-specific cross-neutralization titers. Isolates are represented by colored spheres with antisera shown in gray cubes. Data adapted from Hoshino and colleagues (Hoshino et al., 2004). Grid lines represent a two-fold change in dilution of the plaque reduction neutralization titer. Strain names are colored according to their antigenic cluster as defined by K-means cluster analysis.

We identified three major antigenic clusters within the human and bovine strains, based on previously determined cross-neutralization titers (Hoshino et al., 2004), using k-means clustering (Figure 1). The G9 strains split into two clusters (orange and green cluster strains) and the UK G6 strain (blue cluster strain) fell by itself on the map. Between the green and orange G9 groups, 11 CDMs were identified (V9I, I17V, V44A, A87T, A186S, T208I, A220T, T242N, R250K, E267D, and I287V) (Table 1). Of these, only residues 87, 208, 220, and 242 are surface-exposed. K-means cluster analysis based on pairwise amino acid differences was used to determine whether genetic content could be predictive of antigenic clusters. Hereafter, we refer to clusters based on pairwise amino acid differences as putative antigenic clusters, and the clusters based on cross-neutralization titers (Hoshino et al., 2004) and antigenic cartography as known antigenic clusters.

**TABLE 1:** Cluster defining amino acid mutations between antigenic clusters of RVA G9 VP7 strains^∧^.

Residue	Green cluster	Orange cluster
9	V	I
17	I	V
44	V	A
87*	A	T
208*	T	I
220*	A	T
242*	T	N
250	R	K
267	E	D
287	I	V

^∧^As shown in Figure 1. *Surface-exposed mutation.

**FIGURE 2:**
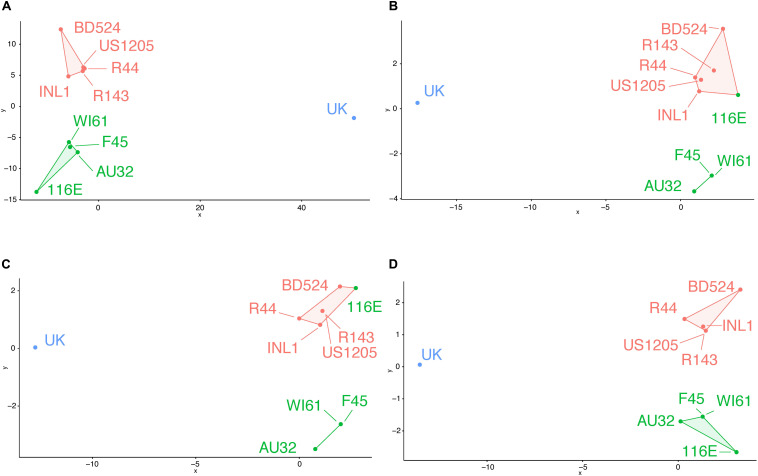
Genetic cluster analysis of RVA VP7 genes using pairwise amino acid differences at variable regions **(A)**, surface exposed variable regions **(B)**, BCEs predicted using an antibody-bound VP7 structure for homology modeling **(C)**, or BCEs predicted using an unbound VP7 structure for homology modeling **(D)**. Strain names are colored according to their antigenic cluster as defined in Figure 1.

Using all variable residues on the VP7 gene, three putative antigenic clusters matched the three known antigenic clusters (Figure 2A). Using all the surface-exposed residues, 116E was incorrectly grouped with the orange antigenic cluster (Figure 2B). The remaining green cluster strains F45, AU32, and WI61 were still grouped together as a separate putative antigenic cluster. Then, BCEs were predicted first using an antibody-bound structure of VP7 as a protein modeling template. Strain 116E was again incorrectly clustered with the orange antigenic group (Figure 2C). Next, BCEs were predicted using the unbound VP7 structure as a modeling template. Several implausible pBCEs were identified on the N-terminus tail between residues 60 and 71, which were excluded from the cluster analysis due to being inaccessible to antibodies. This time, the putative antigenic clusters using the unbound VP7 structure matched the known antigenic clusters (Figure 2D), suggesting the unbound VP7 structure is the better template for homology modeling and generating antigenically relevant pBCEs.

**FIGURE 3:**
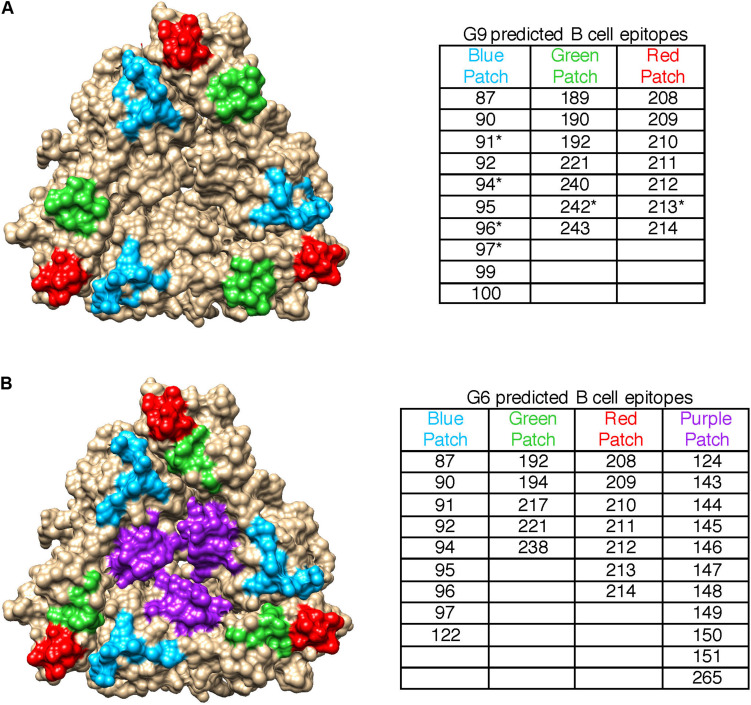
Spatial relationships of VP7 pBCEs. pBCEs are shown on a trimer structure of RVA VP7 (PDB #4v7q) for human G9 RVA **(A)** and bovine G6 RVA **(B)**. pBCEs are colored according to patches. The amino acid residues within each patch are listed in the table, with asterisks indicating pBCEs that are known neutralization escape mutations within human RVA strains WI61, AU32, and F45. No known neutralization escape mutations are known for the remaining human and bovine RVA strains.

Since heterotypic immunity is less robust than homotypic immunity, we compared pBCEs between the G9 and G6 strains to identify genotype-specific patterns in putative epitopes. The G9 pBCEs predicted using the unbound VP7 modeling template fell into three patches located at the interface of VP7 monomers (Figure 3A). The G6 and G9 strains shared 17 pBCEs, but the G6 had an additional patch of pBCEs located in the center of the trimer that was not identified in the G9 strains (Figure 3B). Of the strains included in the PRN experiments performed by Hoshino and colleagues, neutralization escape mutations have only been characterized in WI61, F45 and AU32 (Kirkwood et al., 1993). Neutralization escape mutations at residues 91, 94, 96, 97, 213, and 242 have been reported in these strains and caused resistance to neutralization by mAbs (Kirkwood et al., 1993). All six residues were also pBCEs. Three of the cluster defining mutations in G9 (87, 208, and 242) were pBCEs, and one CDM (220) was next to a pBCE at site 221. The overlap between pBCEs, CDMs, and known neutralization escape mutations (NEMs) overall shows that epitope prediction identifies sites with known functional relevance for conferring neutralization resistance and sites that drive separation between antigenically distant strains.

### Prediction of Porcine RVA VP7 B Cell Epitopes

After validating the BCE prediction pipeline and confirming the biological relevance of pBCEs, we predicted BCEs on 196 porcine RVA VP7 sequences obtained in the United States between 2009 and 2014. About half of the samples were G9 (*n* = 93 sequences), and G3, G4, G5, and G11 genotypes were also observed (Table 2). Mixed genotype infections (G4/G9 or G9/G11) were observed in 3 samples (Table 2).

**TABLE 2:** Distribution of RVA G genotypes in US swine farms between 2009–2011.

Genotype	G3	G4	G5	G9	G11	G4/G9	G9/G11	Total
Number of samples	40	34	17	93	6	2	1	**193**
Percent	20.7%	17.6%	8.8%	48.2%	3.1%	1.0%	0.5%	

**FIGURE 4:**
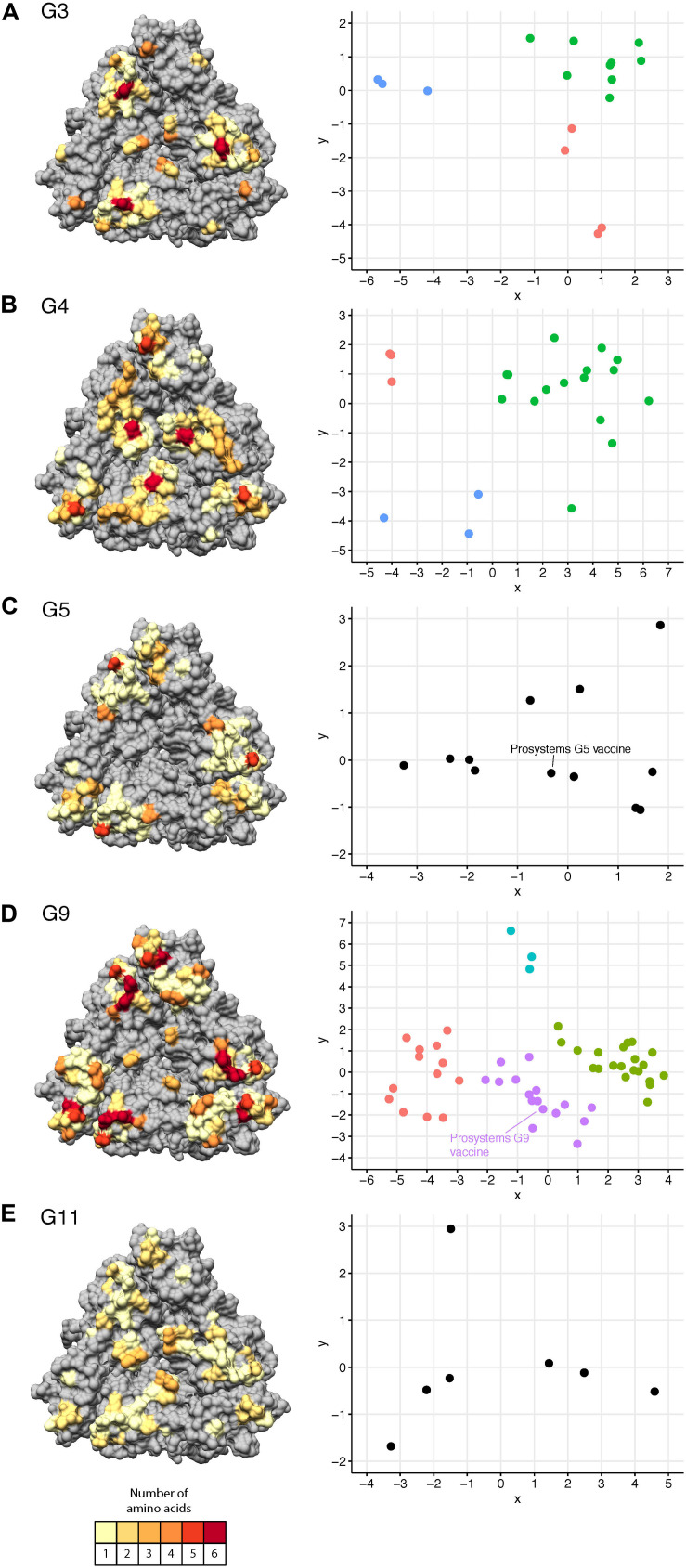
Predicted B cell epitopes and putative antigenic clusters of porcine RVA VP7 genotypes. Left: Genotype-specific pBCEs on a VP7 trimer, colored according to the number of amino acids observed at each position within the respective genotype; G3 **(A)**, G4 **(B)**, G5 **(C)**, G9 **(D)**, and G11 **(E)**. Gray residues: non-pBCEs. Right: Genotype specific putative antigenic clusters of porcine VP7 strains. Colors represent putative clusters, based on K-means cluster analysis. G5 **(C)** and G11 **(E)** are not clustered due to the low sample size of sequences in these genotypes.

Protein modeling of genotype-specific pBCEs illustrated distinctive spatial patterns across genotypes (Figure 4) similar to our observations with the human G9 and bovine G6 dataset (Figures 3A,B). pBCEs unique to individual genotypes included amino acid residues at 86, 123–124, 143–144, and 152 in G4’s; 103, 119, and 216 in G5’s; 189–190, 208–209, and 221–222 in G9s; and 126 in G11’s (Supplementary Figure 2). Additionally, several clusters of pBCEs were found in all the genotypes analyzed including amino acids 87–99, 122, 147, and 210–213 (Supplementary Figure 2). These patches of pBCEs often exhibited higher amino acid diversity and could be key targets of porcine antibodies where high variability would contribute to virus immune escape.

We compared genotype-specific pBCEs to the experimental epitope data available from studies of porcine RVA (Coulson and Kirkwood, 1991; Kang et al., 1993; Kirkwood et al., 1993; Ciarlet et al., 1994) to determine whether pBCEs had any known functional significance. Nine neutralization escape mutations are known in porcine RVA, and all except residue 96 in G4 and 223 in G11 were also pBCEs, highlighting the ability of EPCES to predict sites that interact with antibodies (Supplementary Figure 2). Notably, limited amino acid diversity was present in many sites that are both NEMs and pBCEs, except for site 96 in G5 strains which had 5 possible amino acid residues in our dataset.

### Identification of Putative Antigenic Clusters of Porcine RVA VP7 and Comparison With Vaccine Strains

We used pBCEs from the porcine RVA strains to explore whether putative antigenic clusters are present within the viral genotypes infecting pigs (Figure 4). The G5 and G9 strains from the Prosystem RCE vaccine were included to predict antigenic separation between strains in our dataset and the vaccine. The Prosystem G5 strain was relatively centrally located within pig G5 sequences (Figure 4C). It was most distant from strain Minnesota15, with 4 amino acid changes. The most amino acid changes observed between any two porcine G5 strains was 6. Putative antigenic clusters of G5 strains were not determined due to low sample size.

Four putative antigenic clusters were present within G9 porcine RVA (Figure 4D), and up to nine amino acid changes were observed between the Prosystem G9 strain and porcine G9 strains Iowa148, Iowa149, Illinois96 and Illinois142 (Supplementary Table 2). A maximum of 12 amino acid changes was observed for any G9 strains, indicating greater antigenic separation in porcine G9 RVA compared to G5. CDMs for G9 strains were present at positions 147, 211, 220, and 221 (Table 3 and Supplementary Table 2). Three putative antigenic clusters were predicted in G3 and G4 genotypes (Figures 4A,B) with between 1 and 7 CDMs identified (Table 3 and Supplementary Tables 3, 4). The G11 strains were closely related in shape space indicating low potential antigenic diversity (Figure 4E), but cluster analysis was not performed due to low sample size.

**TABLE 3:** Cluster-defining mutations between putative antigenic clusters of porcine RVA.

Cluster comparison	Site	Amino acid change

G3		
Orange → Green	146	A → T
	213	S → T
Orange → Blue	87	V → T
	92	E → Q
	96	D → N
	122	T → A
	213	S → N
Green → Blue	87	V → T
	91	R → T
	92	E → Q
	96	D → N
	122	T → A
	146	T → A
	213	T → N

**G4**		

Orange → Green	124	I → V
	152	V → I
	211	D → N
Green → Blue	124	V → I
Orange → Blue	152	V → I
	212	T → I

**G9**		

Orange → Green	None identified	
Green → Blue	220	T → K
Blue → Purple	147	K → T
	211	D → N
	221	D → N
Orange → Blue	220	T → K
	221	N → D
Orange → Purple	220	T → K
Green → Purple	211	D → N
	220	T → K

## Discussion

Reducing the burden of rotavirus disease in piglets will require vaccines that cover the antigenic diversity of strains on farms. B cell epitopes can be used to create targeted vaccines and understand whether genetic changes that arise in circulating strains could reduce vaccine efficacy, but little information on BCEs within swine RVs is available. Instead of using cell culture-based methods to identify BCEs, we applied an *in silico* approach to predict BCEs on a dataset of 196 porcine RVA VP7 sequences in order to describe antigenically relevant sites for understanding RVA antigenic diversity on farms. Given the importance of genotype-specific immunity for RVA in the field, we chose to group the strains by genotype to identify common epitopes for strains sharing the same antigenic profile.

Using antigenic cartography to define antigenic clusters within previously characterized RVA strains and comparing the clusters with putative antigenic clusters defined using pBCEs, our research suggests porcine RVA pBCEs have functional relevance for antigenic diversity. Antigenic cartography has been used to model the antigenic relationships among influenza A viruses (de Jong et al., 2007; Lewis et al., 2011, 2014; Lorusso et al., 2011; Abente et al., 2016), dengue viruses (Katzelnick et al., 2015), and lyssaviruses (Horton et al., 2010), but has not yet been applied to rotaviruses due in part to the difficulty of adapting multiple RVA genotypes to culture for cross-neutralization assays which are necessary to define known antigenic clusters. Our results showed that antigenic relationships among RVA strains could be predicted in the absence of cell culture-based approaches. The approach performed well for VP7 epitope prediction, and will next be applied to the VP4 protein since protection *in vivo* is mediated by both VP7 and VP4.

An important finding was that consideration of all surface-exposed residues, and therefore all theoretical antibody binding sites, led to incorrect putative antigenic clusters (Figure 2B). This shows that BCE prediction can help highlight the particular surface-exposed residues on RVA VP7 that are most important for driving antigenic disparities among strains and updating a farm-specific RP vaccine. However, in all comparisons between known and putative antigenic clusters, inter-antigenic groupings were well-recapitulated while intra-antigenic clusters were not always accurate. For instance, WI61 and F45 fell within the same antigenic cluster but were still separated from each other on the antigenic cartography map (Figure 1). In the putative antigenic clusters determined using surface exposed variable residues and pBCEs, they were situated in the same location (Figures 2B–D). Additionally, there is greater antigenic diversity predicted among the orange strains in the putative antigenic clusters than in the known antigenic clusters (Figures 1, 2). These discrepancies indicate that while epitope prediction can help define clusters of antigenically related strains, there is still remaining work on how to bioinformatically predict the finer nuances of cross-neutralization and cross-protection.

Previous studies have drawn conclusions on the efficacy of RVA vaccines against viral diversity within and across genotypes by investigating residue changes at three proposed RVA VP7 epitopes 7-1a, 7-1b, and 7-2 (Zeller et al., 2012; Naseer et al., 2017). These epitopes are a combination of NEMs and sites that are conserved within, but not across, viral genotypes (Aoki et al., 2009; McDonald et al., 2009). Several pBCEs between amino acids 87–99 and 122, 145–147, and 208–214 were consistently predicted across multiple genotypes and correspond to a subset of residues within epitopes 7-1a, 7-2, and 7-1b, respectively. We also found pBCEs unique to individual genotypes which may contribute to genotype-specific patterns of neutralization. This agrees with a previous study indicating that humans generate some VP7-specific mAbs which neutralize heterotypic RVA strains while other mAbs neutralize homotypic RVA (Nair et al., 2017). Use of the mAbs to generate escape mutants showed variants with mutations at different locations, suggesting they target different locations on the capsid protein. Future work is needed to study the antibody specificity of pigs in response to RVA infections to fully elucidate the location of epitopes that are genotype-specific and cross-reactive.

Low amino acid diversity at several NEMs on porcine RVA may highlight differences between immunological responses of pigs and other animal models upon which the existing RVA epitope mapping literature is based (Coulson and Kirkwood, 1991; Kang et al., 1993; Kirkwood et al., 1993; Ciarlet et al., 1994). Research into other porcine pathogens such as porcine respiratory and reproductive virus (PRRSV) has shown that epitopes identified using mAbs from mice are not reactive against PRRSV-positive pig serum, indicating antibody responses can be species-specific (Wang et al., 2014). Therefore, *in silico* methods can predict RVA epitopes that are likely to participate in antibody-antigen interactions, but a limited number of the sites may be antigenic in pigs. Indeed, over 131 epitopes have been proposed in the influenza A virus (IAV) literature, but amino acid substitutions in as few as six residues can result in antigenic changes in porcine H3 IAV (Wiley et al., 1981; Wilson and Cox, 1990; Koel et al., 2013, 2014; Abente et al., 2016). While the remaining proposed IAV epitopes could be functional, evolutionary pressure has likely constrained IAV immune escape to only a few key positions (Koel et al., 2013). A similar phenomenon may occur in RV as well. Studies validating pBCEs in porcine RVA will identify the sites driving immune escape, but we hypothesize that higher genetic variability at pBCEs may be indicative of immunologically important porcine RVA BCEs.

Finally, it is important to recognize the limitations of any *in silico* approach to epitope prediction. EPCES has a reported area under the curve (AUC) value of 0.632, 47.8% sensitivity, and 67.5% specificity at the cutoff which was used in our analysis (Liang et al., 2009). These statistics indicate that epitope prediction cannot be the sole method of understanding antigenically relevant sites, and that further work will be necessary to fully develop this line of research for porcine RVA. While EPCES was able to predict known epitopes of porcine RVA with success, many more BCEs were predicted than have known function and it is highly unlikely that all are true epitopes. Also, while the pBCEs may have all the biochemical and structural components of a BCE, their role in protection *in vivo* depends on whether the pig immune system targets them in the normal course of infection. Thus, this work should be interpreted as a starting point for developing this line of research, and we maintain that the sites here should be confirmed with targeted binding and neutralization experiments that can be performed in the future.

In conclusion, pBCEs enhance our predictions of antigenic distance between field strains using cluster analysis based on pBCE genetic content and could provide a valuable tool to interpret the effect of point mutations on vaccine coverage. While any *in silico* approach has its limitations, this work nonetheless provides a starting point for sites that should be prioritized in future confirmatory work. In addition, mapping RVA strain relationships in space is a quick way to theoretically assess overall antigenic diversity present on a farm, giving veterinarians tools for understanding newly emergent RVA strains on farms in a digestible format. Epitope prediction gives us the ability to study rotaviruses without an established culture system, such as for RVB and RVC, and can lead to enhanced vaccination strategies based on predicted B cell epitopes, thus reducing piglet mortality from all clinically relevant rotavirus species.

## Data Availability Statement

The datasets presented in this study can be found in online repositories. The names of the repository/repositories and accession number(s) can be found below: https://www.ncbi.nlm.nih.gov/genbank/, MN862084–MN862279.

## Author Contributions

FS and DM conceived of and designed the study. FS carried out the analysis and interpreted the results. CD, DM, and MM helped in interpreting the results. FS wrote the manuscript, with feedback and approval by CD and DM. All authors contributed to the article and approved the submitted version.

## Conflict of Interest

The authors declare that the research was conducted in the absence of any commercial or financial relationships that could be construed as a potential conflict of interest.

## Footnotes

1 https://www.geneious.com
