# Supplementary material for: Leveraging a Validated in silico Approach to Elucidate Genotype-Specific VP7 Epitopes and Antigenic Relationships of Porcine Rotavirus A
Source: Front Genet. 2020 Jul 31;11:828. doi: 10.3389/fgene.2020.00828 (PMC7411229; doi:10.3389/fgene.2020.00828)
Supplement: Supplementary file 1 [file Image_1.PDF]

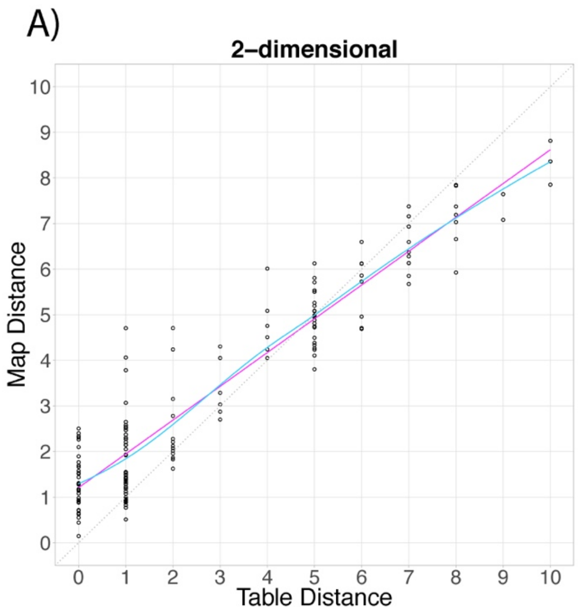

Figure S1. Antigenic cartography table versus map distances in 2 dimensions **(A)** versus 3 dimensions **(B)**. Table distances are measured from the neutralization titers with the equation $\log_2(b_j)-\log_2(N_{ij})$ with b_j indicating the "minimum column basis," automatically adjusted in acmacs-web.antigenic-cartography.org, and N_{ij} representing the titer differences between serum *i* and antigen *j*. Linear regression (pink), loess regression (blue, 0.75 span, 2nd degree polynomial) and perfect fit (dotted gray) lines are shown.
