# Supplementary material for: Leveraging a Validated in silico Approach to Elucidate Genotype-Specific VP7 Epitopes and Antigenic Relationships of Porcine Rotavirus A
Source: Front Genet. 2020 Jul 31;11:828. doi: 10.3389/fgene.2020.00828 (PMC7411229; doi:10.3389/fgene.2020.00828)
Supplement: Supplementary file 2 [file Image_2.pdf]

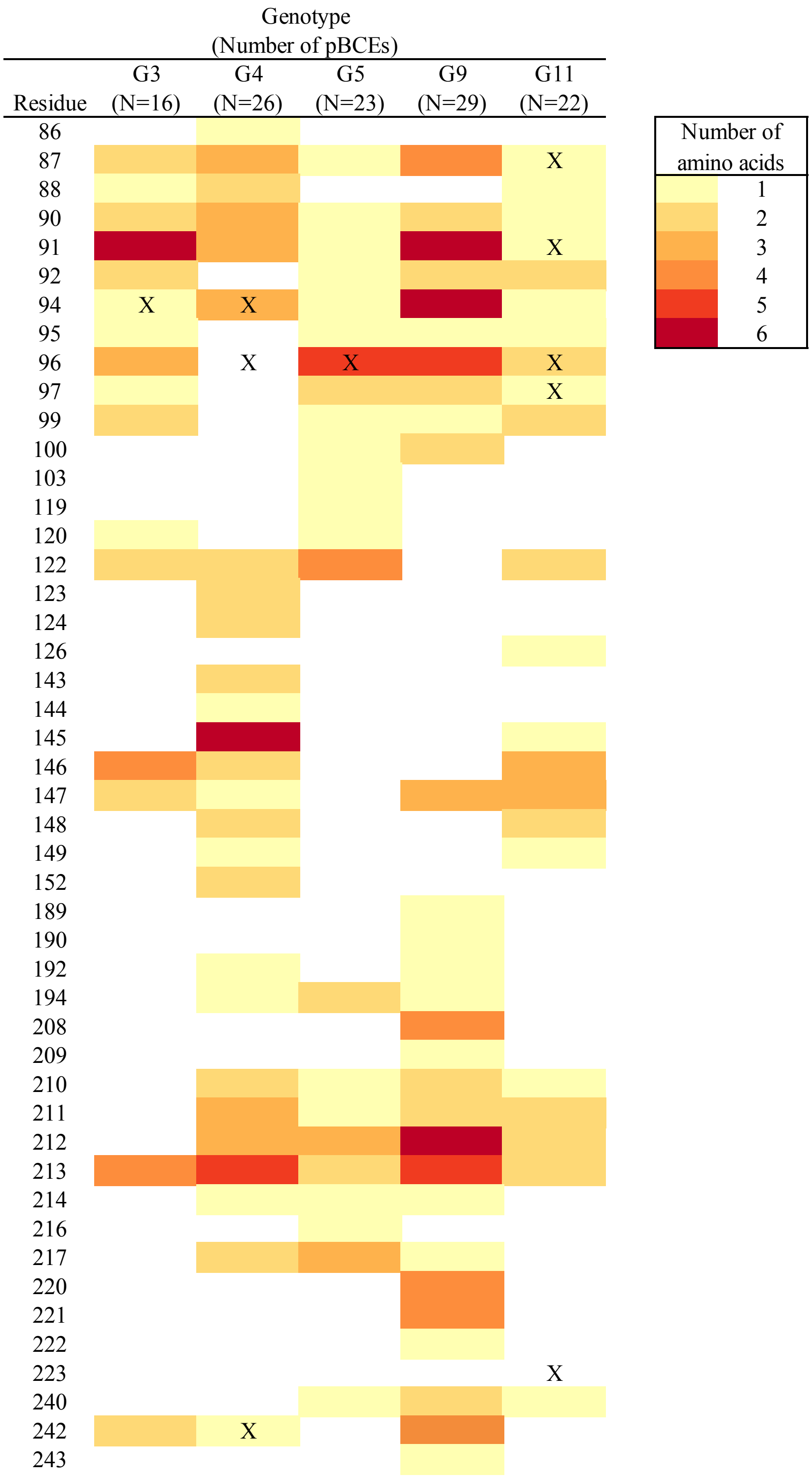

Figure S2. Amino acid diversity and neutralization escape mutations at predicted B cell epitopes. Porcine RVA VP7 genotype-specific pBCEs were predicted and compared with in vitro neutralization escape mutations (NEMs). Shading of a box indicates the residue is a pBCE and is colored according to number of amino acids present in the alignment. X indicates the site is a porcine RVA NEM.
