# Supplementary material for: Leveraging a Validated in silico Approach to Elucidate Genotype-Specific VP7 Epitopes and Antigenic Relationships of Porcine Rotavirus A
Source: Front Genet. 2020 Jul 31;11:828. doi: 10.3389/fgene.2020.00828 (PMC7411229; doi:10.3389/fgene.2020.00828)
Supplement: Supplementary file 3 [file Table_1.DOCX]

**Table S1**. Stress reduction and goodness of fit in 2D versus 3D antigenic cartography.

|  | 2 dimensions | 3 dimensions | 4 dimensions |
| --- | --- | --- | --- |
| Raw stress value | 252.5465 | 252.3915 | 252.3915 |
| Percent reduction |  | 0.06%  (vs 2-dimensions) | 0 %  (vs 3-dimensions) |
| Linear regression equation |  |  |  |
| Slope | 0.74 | 0.74 | 0.74 |
| Intercept | 1.21 | 1.21 | 1.21 |
| R^2^ | 0.89 | 0.90 | 0.90 |
| Adjusted R^2^ | 0.89 | 0.90 | 0.90 |
